# Neuroprotective Effects of Trimetazidine against Cisplatin-Induced Peripheral Neuropathy: Involvement of AMPK-Mediated PI3K/mTOR, Nrf2, and NF-*κ*B Signaling Axes

**DOI:** 10.1155/2024/6612009

**Published:** 2024-08-20

**Authors:** Marawan A. Elbaset, Sherif M. Afifi, Tuba Esatbeyoglu, Sahar S. Abdelrahman, Dalia O. Saleh

**Affiliations:** ^1^ Pharmacology Department Medical Research and Clinical Studies Institute National Research Centre, 33 El-Bohouth Street, Dokki, Cairo P.O. 12622, Egypt; ^2^ Department for Life Quality Studies Rimini Campus University of Bologna, Corso d'Augusto, 237, Rimini 47921, Italy; ^3^ Department of Molecular Food Chemistry and Food Development Institute of Food and One Health Gottfried Wilhelm Leibniz University, Am Kleinen Felde 30, Hannover 30167, Germany; ^4^ Department of Pathology College of Veterinary Medicine Cairo University, Cairo P.O. 12211, Egypt

## Abstract

Cisplatin-induced peripheral neuropathy (CIPN) is a common and debilitating side effect of cisplatin chemotherapy used in cancer treatment. This study explored the neuroprotective effects of Trimetazidine (TRI) against CIPN by preserving nerve integrity, reducing neuro-oxidative stress, and alleviating neuroinflammation. Using a rat model of CIPN, we evaluated TRI's impact on motor coordination, pain sensitivity, and peripheral nerve histopathology. Also, its effects on neuro-oxidative stress and neuroinflammatory markers were assessed. The findings showed that rats with CIPN had worse motor coordination and increased sensitivity to pain but that these symptoms were alleviated by TRI therapy in a dose-dependent way. Nerve conduction velocities were normalized, and expression of genes involved in neuropathy signaling was suppressed after TRI therapy. Antioxidant benefits were also shown in TRI, with oxidative damage being reduced and the cellular energy balance being restored. By inhibiting the production of inflammatory markers, it also demonstrated anti-inflammatory properties. Histopathological examination revealed that TRI, especially when administered at a higher dose, inhibited the degeneration and demyelination of nerve fibers. The anti-inflammatory properties of TRI in the sciatic nerves were further shown by the fact that its administration reduced iNOS expression. In conclusion, AMPK-mediated PI3K/mTOR, Nrf2, and NF-*κ*B signaling pathways may all be involved in the therapeutic benefits of TRI for CIPN. These results indicate that TRI may be useful for reducing the side effects of CIPN and enhancing patient outcomes during cisplatin chemotherapy.

## 1. Introduction

Damage to the peripheral nervous system causes the symptoms of peripheral neuropathy (PN), a common neurological illness that may impair sensory, motor, and autonomic neurons [1]. Several chemotherapeutic agents are employed in cancer treatment, among which cisplatin (Cis) is notable. However, cisplatin is known to induce severe side effects [2]. The chemotherapeutic medication cis, which is based on the metal platinum, is frequently used because of its effective anticancer characteristics. However, the emergence of PN often impedes its therapeutic usage, diminishing both the quality of life and treatment results for cancer patients [3].

Cisplatin exerts its antitumor effects by forming DNA adducts, causing cross-linking and subsequent cellular apoptosis [4]. Unfortunately, this drug also targets healthy cells, leading to collateral damage in peripheral nerves. The precise mechanisms underlying cisplatin-induced peripheral neuropathy (CIPN) remain multifaceted and poorly understood [5]. However, current research efforts have shed light on several key aspects contributing to this neurotoxicity. Understanding the molecular mechanisms underlying CIPN is vital for developing effective therapeutic interventions. Various strategies, including antioxidant agents, neuroprotective compounds, and anti-inflammatory drugs, have been investigated to mitigate or prevent CIS-induced neurotoxicity [6].

CIPN represents a significant clinical challenge in cancer therapy. Elucidating the intricate mechanisms driving this neurotoxicity will enable the development of targeted preventive and therapeutic strategies [7]. By comprehensively understanding the impact of CIS on PN, we can alleviate treatment-associated morbidity and enhance the overall well-being of cancer patients, thereby improving the success of chemotherapy interventions [8]. The anticancer properties of CIS have been well-documented; however, it is also associated with severe neurotoxicity. Studying the effects of CIS on rats' motor coordination and nociceptive thresholds is crucial for deepening our understanding of its negative consequences.

Usually, TRI (1-(2,3,4-trimethoxybenzyl) piperazine dihydrochloride) is often used for the treatment of ischemia [9]. It reduces oxygen use by switching the body's fuel source from fat to sugar [10]. Apart from its cardioprotective effects, there is emerging evidence suggesting the potential positive effects of TRI on neuropathic disorders. Studies have indicated that TRI may have neuroprotective properties, which could potentially be beneficial for neuropathy [11]. One proposed mechanism of action is its ability to improve cellular energy metabolism. By optimizing energy production in nerve cells, TRI may help reduce oxidative stress and prevent damage to the nerves. It has been suggested that this metabolic modulation could potentially slow down the progression of neuropathy and alleviate symptoms [12].

The findings of this study highlight the beneficial effects of TRI across various parameters, including motor coordination, pain sensitivity, nerve conduction velocities, gene expression, oxidative stress levels, cellular energy metabolism, inflammatory markers, histopathological changes, and iNOS expression in CIS-induced peripheral neuropathic rats. Overall, this research enhances our understanding of TRI's therapeutic potential in alleviating CIPN and offers valuable insights for developing new strategies to manage chemotherapy-induced neuropathy.

## 2. Materials and Methods

### 2.1. Animals

Wistar rats weighing (175–225 g) of male gender were brought from the “National Research Centre (Giza, Egypt). In the animal house of the Faculty of Pharmacy, Cairo University (Cairo, Egypt).” The animals were kept for a week so they could get used to their surroundings. They were acclimated in an ambient condition with an equal day-to-night. The rats had free access to tap water and regular lab food. The investigations were carried out in compliance with “the National Institutes of Health's ethical standards for the care, use, and handling of laboratory animals as per the Ethics Committee of the NRC (24112012023) and followed the recommendations of the National Institutes of Health Guide for Care and Use of Laboratory Animals (NIH Publication No. 85-23, revised).”

### 2.2. Drugs and Chemicals

Trimetazidine (TRI) was acquired from Global Napi (Cairo, Egypt), while Cisplatin (Cis) was supplied by the Mylan SAS pharmaceutical company (Saint-Priest, France). All other chemicals used in the studies were of the highest purity and analytical grade. Doses of 20 or 40 mg/kg/day of TRI that had been freshly suspended in a 1% tween 80 solution were orally given to rats [13]. Simultaneously, Cis (2 mg/kg) was injected twice weekly for 6 weeks [14].

### 2.3. Induction of Peripheral Neuropathy

The rats were given Cis (2 mg/kg) intraperitoneally (ip) twice weekly for 6 weeks to produce neuropathy.

### 2.4. Experimental Design

In the initial trial, a total of 54 rats were assigned randomly to four groups, with 18 rats in each group. Group I, the control group, received daily oral administration of a 1% tween 80 solution and intraperitoneal saline injections twice a week for 6 weeks. Group II consisted of rats who received daily oral administration of a 1% tween 80 solution and were given Cis (2 mg/kg) ip twice weekly for 6 weeks and served as the neuropathic Cis group. Meanwhile, groups III and IV, also neuropathic rats, were treated with daily oral doses of TRI at 20 or 40 mg/kg dissolved in 1% tween 80 solutions for 6 weeks and given Cis (2 mg/kg) ip twice weekly for 6 weeks.

As per Abdelkader et al. [15, 16] 1 day following the last medication administration, all rats were subjected to a series of behavioral tests, progressing from the least painful (rotarod) to the most distressing (Randell–Selitto, cold allodynia of the hind paws and hot plate). Experiments were repeated twice, with a 1-hr gap in between, in a soundproof room throughout the day [15, 16]. Six rats from each group were anesthetized with ketamine. They were sedated by intraperitoneal injection of xylazine and ketamine solution (20 and 50 mg/kg, respectively). Following this, they underwent electrophysiological testing of their right sciatic nerves 1 day after the last administration of medication. Subsequently, the rats were euthanized, and their sciatic nerves were not utilized for further analysis.

Following euthanasia of the remaining rats under anesthesia, six randomly selected rats per group had their right sciatic nerves dissected, cleaned, frozen in liquid nitrogen, and stored at −80°C until they were used for quantitative real-time PCR analysis of AMPK, mTOR, and PI3K. Inducible nitric oxide synthase (iNOS) was quantified through immunohistochemistry after the left sciatic nerve was fixed in 10% neutral-buffered formalin overnight. Fast dissection, rinsing in ice-cold saline, and homogenization in phosphate buffer were performed on the remaining six rats per group for both left and right sciatic nerves. The homogenates were centrifuged at 15,000 *g* for 15 min at 4°C to separate them. Aliquots of the supernatants were frozen at 80°C for later use to estimate the biochemical parameters; malondialdehyde (MDA), nitric oxide (NO), adenosine triphosphate (ATP), adenosine diphosphate (ADP), nuclear factor erythroid 2–related factor 2 (Nrf2), NADPH oxidase 1 (NADPH), nuclear factor kappa B (NF*κ*B) and phospho-nuclear factor kappa B P65 (*p*-NF*κ*B) [15, 16].

### 2.5. Behavioral Analysis

#### 2.5.1. Rotarod Test

Motor coordination was assessed following the method described by Ogaly et al. [17] using the Ugo Basile accelerated rotarod (Model 47750, Varese, Italy). Rats were subjected to an accelerating wheel ranging from 4 to 40 rounds/min for 5 min, and the duration each rat remained on the rod before falling off was recorded.

#### 2.5.2. Randell–Selitto Test

The sensitivity to pressure force was assessed utilizing the Ugo Basile analgesia meter (Model 7200, Varese, Italy), following the methodology described by Leighton et al. [18]. The paw withdrawal time was recorded as an indicator of sensitivity to pressure force.

#### 2.5.3. Hind Paw Cold Allodynia Test

The sensitivity to low temperature was evaluated following the protocol outlined by Abdelkader et al. [15, 16] in which an ice-cold temperature of 3–4°C was applied to the paw, and the time taken for paw withdrawal upon contact was recorded [15, 16].

#### 2.5.4. Hot Plate Test

The sensory pain receptor was tested in agreement with Abdelkader et al. [15, 16], in which “Ugo Basile hot plate apparatus (Model 7280, Varese, Italy)” was utilized to record the time for rats to withdraw their hind paw was recorded [15, 16].

### 2.6. Electrophysiology of Sciatic Nerve

The sensory nerve conduction velocity (SNCV) and motor nerve conduction velocity (MNCV) were measured following the method described by Abdelkader et al. [15, 16]. For the experiments, rats were anesthetized using a combination of xylazine and ketamine (20 and 50 mg/kg, i.p., respectively). The Ugo Basil ECT Unit (Model 57800, Italy) was utilized to stimulate the right sciatic nerve with specific parameters: a pulse duration of 0.1 ms, an intensity of 20 mA, and a frequency of 50 Hz. The action potential was then recorded using the PowerLab 8SP system (AD Instruments, Australia) at a frequency of 10 Hz. The SNCV and MNCV were calculated by dividing the distance between distal and proximal cathodes by the latency difference between proximal and distal cathodes [15, 16].

### 2.7. Biochemical Analysis

#### 2.7.1. Quantitative Real-Time RT-PCR Analysis

We extracted total RNA from homogenized tissues of all experimental groups using the Direct-zol RNA Miniprep Plus kit (Catalog No. R2072, ZYMO RESEARCH CORP. USA). Subsequently, they evaluated the quantity and quality of the extracted RNA using a Beckman dual spectrophotometer (USA).

The method of Abdelhameed et al. [19] was adopted in which “SuperScript IV One-Step RT-PCR kit (Cat# 12594100, Thermo Fisher Scientific, Waltham, MA USA) was utilized for reverse transcription of extracted RNA followed by PCR. 96-well plate StepOne instrument (Applied Biosystem, USA) was used in a thermal profile as follows: 10 min at 45°C for reverse transcription, 2 min at 98°C for RT inactivation and initial denaturation by 40 cycles of 10 s at 98°C, 10 s at 55°C and 30 s at 72°C for the amplification step. After the RT-PCR run, the data were expressed in cycle threshold (Ct) for the target genes and housekeeping genes. Normalization for variation in the expression of target genes, AMPK, mTOR, and PI3K, was performed by referring to the mean critical threshold expression values of the GAPDH housekeeping gene by the *ΔΔ*Ct method. The relative quantitation of each target gene is quantified according to the calculation of the 2^−*∆∆*Ct^ method [19]”. Primers sequence for AMPK gene was; forward 5′-CAGCACCGGAGGTCATCTCA-3′, and reverse 5′-GCACGTGCTCATCGTCGAA-3′ Gene bank accession number (NM_023991.1), primers sequence for mTOR gene was; forward 5′-CAGTTCGCCAGTGGACTAAG-3′ and reverse 5′-GCTGGTCATAGAAGCGGAC-3′ Gene bank accession number (XM_021200534.1), primers sequence for PI3K gene was; forward 5′-TCTATGCCTGCTCTGTAGTGG-3′ and reverse 5′-GTGTGA CATTGAGGGAGTCGTTG-3′ Gene bank accession number (XM_032898971.1), and GAPDH housekeeping gene was; forward 5′-GACTTCAACAGCAACTCCCAC-3′ and reverse 5′-TCCACCACCCTGTTGCTGT-3′ Gene bank accession number (XM_039111236.1).

#### 2.7.2. Colorimetric Assay

The levels of malondialdehyde (MDA) and nitric oxide (NO) in sciatic nerve homogenates were measured using commercially available colorimetric kits from Biovision Incorporated (Catalog #: K739-100 and #: K262-200, respectively, Milpitas, CA, USA) as per the kits' instruction manual.

#### 2.7.3. Enzyme-Linked Immunosorbent Assay (ELISA)

Following manufacturer's instructions, rat-specific ELISA kits supplied by Cloud-Clone Corp (Catalog #: CEA349Ge, Katy, USA), Northwest Life Science Specialties (Catalog #: NWK-NRF2R, Vancouver, WA, USA), Abbexa (Catalog #: abx256662 Houston, USA) and (Catalog #: PEL-NFKBP65-S536 RayBiotech, Peachtree Corners, GA, USA) were used to evaluate ATP, Nrf2, NADPH, and *p*-NF*κ*B-P65, respectively. Also, the rat ELISA kit was obtained from MyBioSource (Catalog #: MBS744390 and MBS453975 San Diego, CA, USA) to determine the sciatic content of ADP and NF*κ*B.

#### 2.7.4. Determination of Tissue Protein

Protein content in the tissue was determined per the guide of the protein estimation kit: Genei, Bangalore, India (Catalogue No.: 2624800021730).

### 2.8. Histopathologic Examination

#### 2.8.1. Light Microscopy

After scarification, the sciatic nerves of rats of all groups were dissected. Then, they were fixed in 10% neutral formalin for 1 week, after which they were subjected to a routine histological technique for staining with H&E, according to Bancroft and Gamble [20]. An Olympus 330E microscope was used to look at the slides and take pictures. Neuronal histopathological lesions were counted and semi-quantitatively assessed according to severity and distribution in H&E-stained sections. A score of 0 indicates minimal damage to the sciatic nerve (2%), 1 indicates mild damage (3%–25%), 2 indicates moderate damage (26%–50%), 3 indicates severe damage (51%–75%), and 4 indicates extensive damage (>75%) [21].

#### 2.8.2. Immunohistochemistry

Paraffin slices were microwaved for 25 min at 720 W to retrieve antigens prior to immunohistochemical analysis. The cells were treated at 4°C with a 1 : 200 dilution of a rabbit recombinant multiclonal monoclonal antibody to iNOS (ab283655; Abcam, Cambridge, MA, USA). After being washed in PBS, sections were incubated for 30 min with the biotinylated secondary antibody and streptavidin/Alkaline phosphatase complex at room temperature. The antibody binding sites were seen with DAB (Sigma), washed away with PBS, and then counterstained with Hematoxylin for 2–3 min. The samples were then dehydrated in a series of escalating ethanol solutions, clarified by soaking twice at room temperature xylene for 5 min, mounted, and inspected under a light microscope (Olympus BX50, Japan). We used image analysis software to determine the OD of the positive regions of the marker's expression in 7 high-magnification microscopic areas (Image J, 1.46a, NIH, Bethesda, MD, USA).

### 2.9. Statistical Analysis

Before proceeding with the statistical analysis, data values were checked for normality using the Shapiro test and heteroscedasticity using the Brown–Forsythe test. The data are presented as one-way ANOVA followed by the Tukey processed means ± S.E. Data–Kramer Post hoc test except for the histopathological scoring; statistical analysis was carried out by nonparametric Kruskal–Wallis *H*-test, followed by Dunn's test. GraphPad Prism software (version 9; GraphPad Software, Inc., San Diego, CA, USA) was employed to perform the statistical analysis and to establish the graphical representation.

## 3. Results

### 3.1. Effect of Trimetazidine on Motor Co-ordination and Nociceptive Thresholds in CIS-Induced Peripheral Neuropathic Rats

**Figure 1:**
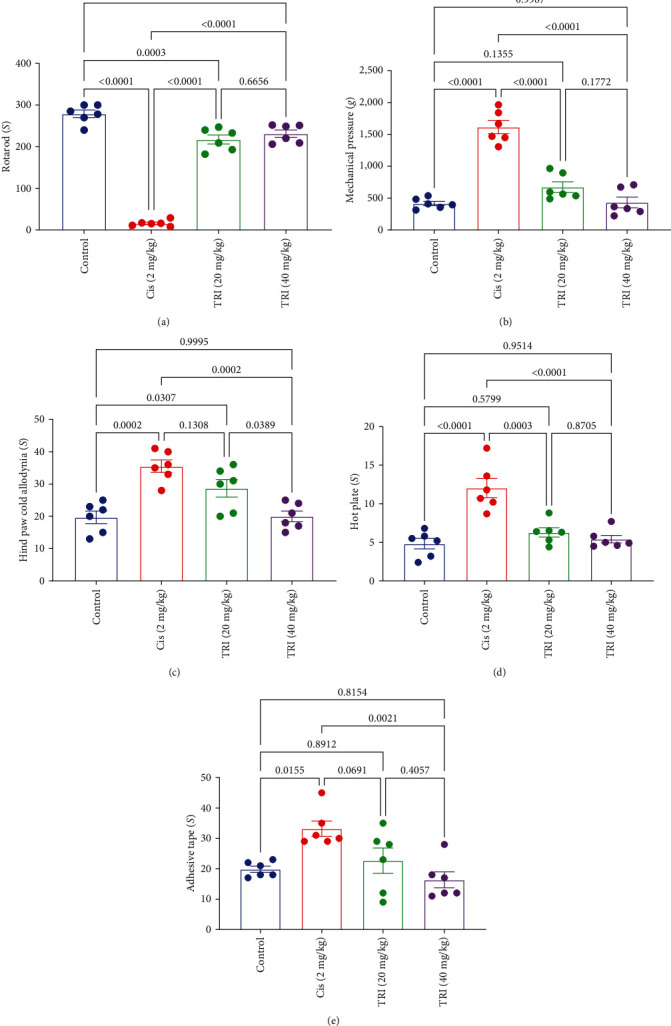
Effect of trimetazidine on motor coordination: (a) rotarod and nociceptive thresholds, (b) mechanical pressure, (c) hind paw cold allodynia, (d) hot plate, and (e) adhesive tape in Cis-induced peripheral neuropathic rats. Each bar represents the mean ± SE of six rats. The values above the pairwise comparison represent the exact *P*-value. TRI, trimetazidine; Cis, cisplatin.

Rats treated with CIS experienced a reduction of the rotarod falling time by 94% compared with the control group. On the other hand, the CIS group exhibited elevation in the threshold time of Hind paw cold allodynia, threshold force of Randall–Selitto mechanical test, reaction time of the hot plate test, and time of adhesive tape removal test by 81%, 290%, 150%, and 67%, in comparison with the control group, respectively (Figure 1).

At the same time, treatment with TRI (20 or 40 mg/kg) increased the falling time of the rotarod by 13.5- and 14.4-fold compared to the control group. Also, TRI (20 or 40 mg/kg) groups exhibited lower threshold force of the Randall–Selitto mechanical test by 58% and 73%, besides shortening the reaction time of the hot plate test by 48% and 55%, respectively. Similarly, only the administration of TRI (40 mg/kg) markedly retracted the threshold time of Hind paw cold allodynia and the time of adhesive tape removal by 44% and 50%, respectively (Figure 1).

### 3.2. Effect of Trimetazidine on Sciatic SNCV and MNCV in CIS-Induced Peripheral Neuropathic Rats

**Figure 2:**
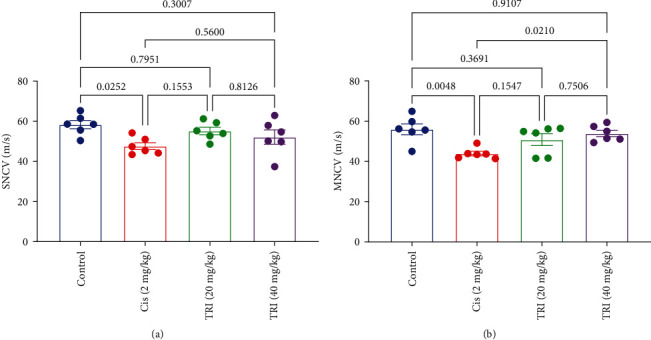
Effect of trimetazidine on sciatic (a) sensory nerve conduction velocity and (b) motor nerve conduction velocity in Cis-induced peripheral neuropathic rats. Each bar represents the mean ± SE of six rats. The values above the pairwise comparison represent the exact *P*-value. TRI, trimetazidine; Cis, cisplatin; SNVC, sensory-motor nerve conduction velocity; MNCV, motor nerve conduction velocity.

In the experimental model of neuropathy, rats with neuropathic symptoms exhibited a significant decrease in SNCV) and MNCV, measuring only 18% and 22%, respectively, when compared to control rats, as shown in Figure 2. This reduction in nerve conduction velocities indicated impaired nerve function in the neuropathic rats.

In contrast, when these neuropathic rats were treated with TRI at doses of 20 or 40 mg/kg, a remarkable improvement in both SNCV and MNCV was observed compared to the group that received only Cis. The TRI, especially the higher dose treatment, effectively restored the nerve conduction velocities, bringing them back to levels similar to those seen in control rats. This indicates that TRI had a positive and dose-dependent effect on the restoration of nerve conduction velocities in the neuropathic rats affected by CIPN (Figure 2).

### 3.3. Effect of Trimetazidine on Sciatic Gene Expression Level of AMPK, mTOR, and PI3K in CIS-Induced Peripheral Neuropathic Rats

**Figure 3:**
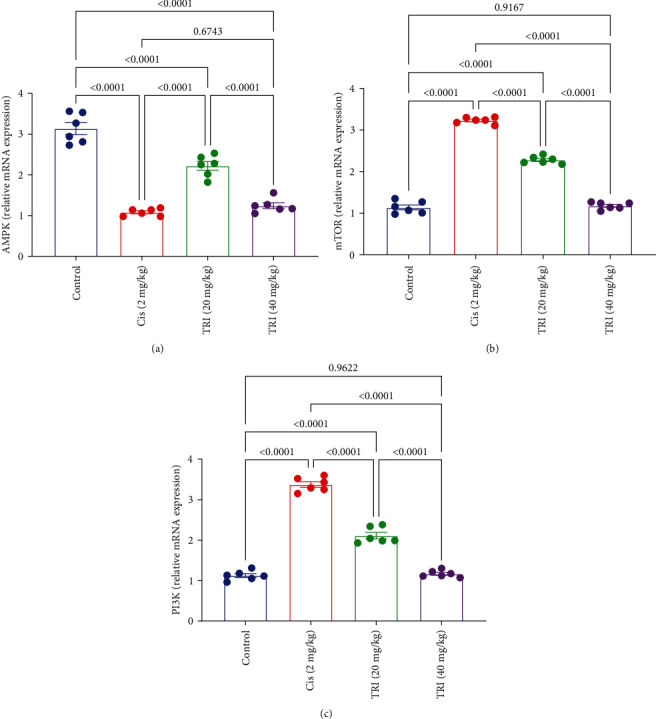
Effect of trimetazidine on sciatic nerve gene expression level of (a) AMPK, (b) mTOR, and (c) PI3K in Cis-induced peripheral neuropathic rats. Each bar represents the mean ± SE of six rats. The values above the pairwise comparison represent the exact *P*-value. TRI, trimetazidine; Cis, cisplatin; AMPK, AMP-activated protein kinase; mTOR, Mammalian target of rapamycin; PI3K, phosphoinositide 3-kinases.

Figure 3 elicited the decline in the gene expression of AMPK in the sciatic nerve associated with CIPN by about 66% compared to the control group. Treatment of CIS-induced peripheral neuropathic rats with TRI (20 or 40 mg/kg) augmented the AMPK gene expression by about 81% and 124%, respectively.

On the other hand, rats given the CIS showed a noticeable upregulation level of the mTOR and PI3K expression by almost three times higher than the control group. However, compared to the CIS group, the TRI (20 or 40 mg/kg) reduced sciatic mTOR levels by 29% and 63% and sciatic PI3K levels by 37% and 65%, respectively (Figure 3).

### 3.4. Effect of Trimetazidine on Sciatic Oxidative Stress Parameters in CIS-Induced Peripheral Neuropathic Rats

**Figure 4:**
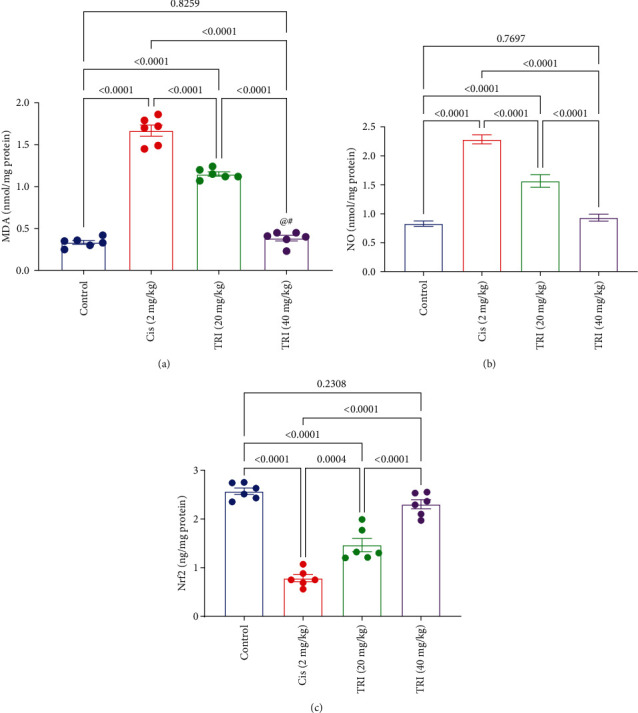
Effect of trimetazidine on sciatic nerve oxidative stress parameters (a) MDA, (b) NO, and (c) Nrf2 in Cis-induced peripheral neuropathic rats. Each bar represents the mean ± SE of six rats. The values above the pairwise comparison represent the exact *P*-value. TRI, trimetazidine; Cis, cisplatin; MDA, malondialdehyde; NO, nitric oxide; Nrf2, nuclear factor erythroid 2–related factor 2.

The rats intoxicated with CIS depicted marked elevation of the sciatic MDA and NO content by about 5- and 2.8-fold, as well as a decline in the sciatic Nrf2 levels by about 69% compared to the control group. On the other hand, the rats that received TRI revealed a noticeable diminution in the sciatic MDA and NO content by 31% and 77% for TRI 20 mg/kg and 31% and 59% for TRI 40 mg/kg compared to the CIS group, respectively. Neuropathic rats treated with TRI (20 and 40 mg/kg) also showed a restoration in the level of Nrf2 by about 1.9- and 2.9-fold compared to the CIS group (Figure 4).

### 3.5. Effect of Trimetazidine on Sciatic Cellular Energy Parameters in CIS-Induced Peripheral Neuropathic Rats

**Figure 5:**
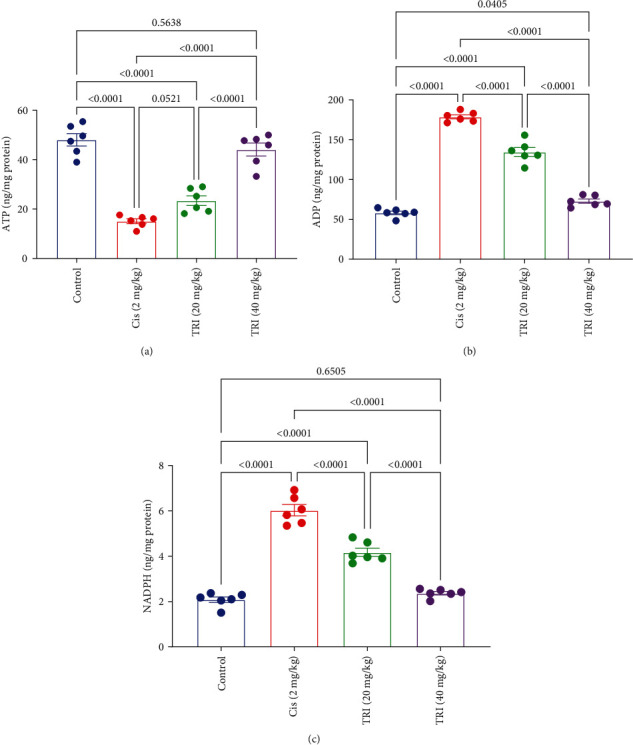
Effect of trimetazidine on sciatic nerve cellular energy parameters (a) ATP, (b) ADP, and (c) NADPH in Cis-induced peripheral neuropathic rats. Each bar represents the mean ± SE of six rats. The values above the pairwise comparison represent the exact *P*-value. TRI, trimetazidine; Cis, cisplatin; ATP, adenosine triphosphate; ADP, adenosine diphosphate; NADPH, nicotinamide adenine dinucleotide phosphate.

At the same time, the sciatic content of the ADP was significantly elevated in the CIS group by threefold in comparison to the control group, while its level was prominently ameliorated in the TRI 20 or 40 mg/kg) group by 24% and 59% compared to the CIS group, respectively. On the contrary, the CIS group's sciatic content of the ATP experienced a marked reduction of 69% compared to the control group. In contrast, only the TRI (40 mg/kg) group sciatic content of the ATP was normalized (Figure 5).

The rats given CIS suffered from elevation in the sciatic nerve content of NADPH by 2.9-fold compared to the control group, respectively. However, the TRI groups declined the sciatic nerve content of NADPH by 31% for the TRI (20 mg/kg), along with 61% for the TRI (40 mg/kg). In contrast, it restored the sciatic nerve content of NADPH to comparable values of the control group (Figure 5).

### 3.6. Effect of Trimetazidine on Sciatic Inflammatory Markers in CIS-Induced Peripheral Neuropathic Rats

**Figure 6:**
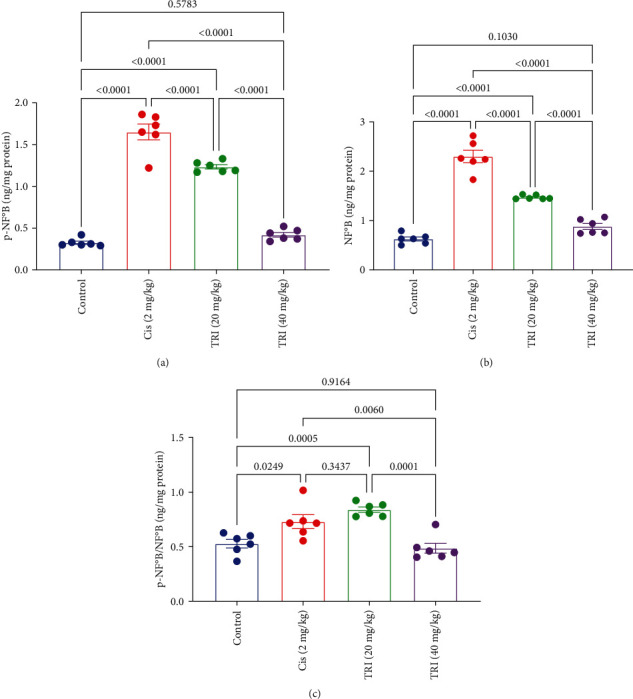
Effect of trimetazidine on sciatic nerve inflammatory markers (a) p-NF-*κ*B, (b) NF-*κ*B, and (c) p-NF-*κ*B/-NF-*κ*B in Cis-induced peripheral neuropathic rats. Each bar represents the mean ± SE of six rats. The values above the pairwise comparison represent the exact *P*-value. TRI, trimetazidine; Cis, cisplatin; *p*-NF-*κ*B, phosphate nuclear factor kappa B, NF-*κ*B, nuclear factor kappa B.

The rats given CIS suffered from elevation in the sciatic nerve content of NF*κ*B and *p*-NF*κ*B-P65 by four- and fivefold compared to the control group, respectively. However, the TRI groups declined the sciatic nerve content of NF*κ*B and *p*-NF*κ*B-P65 by 36% and 25% for the TRI (20 mg/kg), along with 62% and 75% for the TRI (40 mg/kg). Interestingly, the TRI (40 mg/kg) tended to normalize the sciatic nerve content of NF*κ*B and *p*-NF*κ*B-P65 (Figure 6). In the cisplatin group, the marked increase in both p-NF*κ*B-P65 and total NF*κ*B levels suggests an overall upregulation of the NF*κ*B signaling pathway, likely due to the neuroinflammatory and oxidative stress induced by cisplatin. In the TRI treatment groups, the reduction in both p-NF*κ*B-P65 and total NF*κ*B levels suggests that TRI suppresses the overall activation and expression of the NF*κ*B pathway in the sciatic nerves of cisplatin-treated rats.

### 3.7. Effect of Trimetazidine on the Sciatic Nerve Histopathological Alterations in CIS-Induced Peripheral Neuropathic Rats

**Figure 7:**
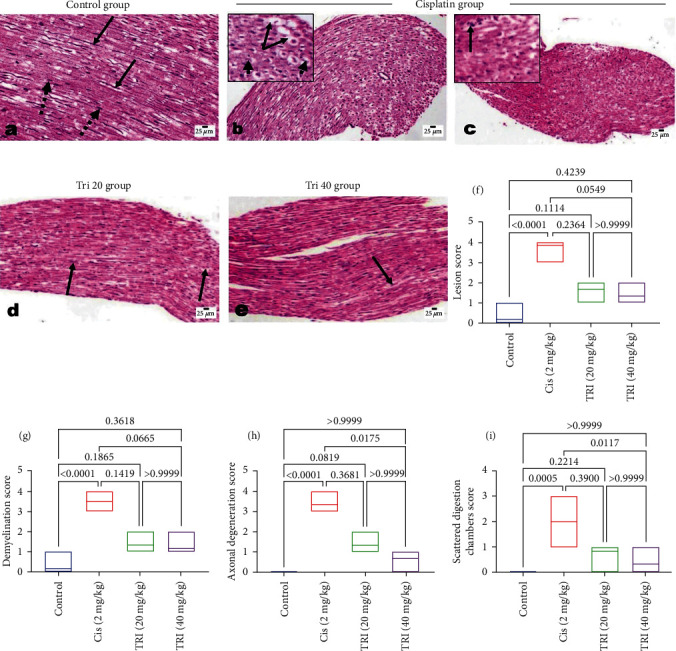
Photomicrograph of H&E-stained sciatic nerve sections of various experimental groups eliciting the effect of trimetazidine on histopathological alterations in Cis-induced peripheral neuropathic rats. (a) The control rat shows normal endoneurium, axons (arrow), and nuclei of Schwann cells (dotted arrow). (b and c) Cisplatin-administered rats showed disorganization of the nerve fibers, segmental demyelination (arrow), swollen axons (arrowhead), and scattered digestion chamber (arrow in c). (d, and e) Tri-administered rats showed dose-related restorative effects with mild axonal degeneration (arrow) and mild demyelination. (f–i) scoring of various histopathological alterations in different experimental groups (Each bar represents the median (interquartile range) of six fields. The values above the pairwise comparison represent the exact *P*-value.

Sections of the sciatic nerve of control rats show normal histological with normal perineurium and endoneurium sheathing each nerve fiber from other fibers. Myeline sheaths are unstained because it is dissolved during tissue processing as it is lipid in nature. Both Schwann cells' nuclei and the central axons are visible (Figure 7(a)). Sciatic nerves of CIS-administrated rats reveal marked PN with loss of the symmetrical appearance of nerve fibers. The nerve fibers are disorganized and degenerated, with segmental demyelination in the form of prevalent vacuolations. Most of the axons appear swollen and degenerated, and few appear pyknotic, with a few scattered digestion chambers (Figures 7(b) and 7(c)). The administration of TRI at both concentrations shows a reasonable degree of therapeutic effect, particularly during the higher dose treatment. Mild to moderate nerve fiber degeneration, mild demyelination, and mild axonal degeneration were prominent, especially at the lower dose (Figures 7(d) and 7(e)).

### 3.8. Effect of Trimetazidine on the Sciatic Nerve iNOS Immunohistochemical Staining in CIS-Induced Peripheral Neuropathic Rats

**Figure 8:**
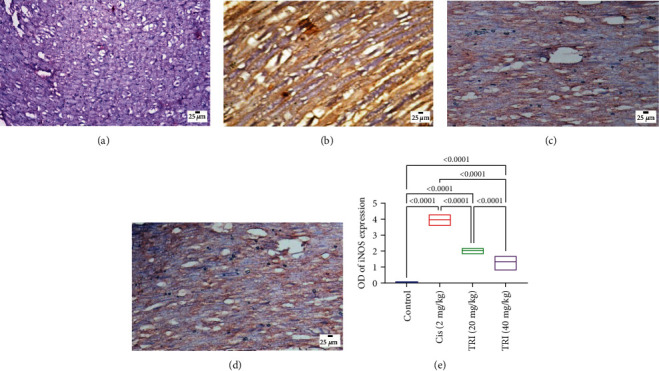
Photomicrographs of immunohistochemical stained sciatic nerve sections for iNOS expression: (a) control group; (b) cisplatin group; (c) Tri 20 group; (d) Tri 40 group; (e) The values above the pairwise comparison represent the exact *P*-value.

Figure 8 represents the immune expression of iNOS in the sciatic nerves of all the experimental groups. The CIS-administered rats show the highest expression of iNOS in their sciatic nerves compared to the control and treated groups. TRI treatment markedly decreased the expression of iNOS in the sciatic nerves of the treated groups, predominantly the higher-dose treated group. Image analysis software performed the quantitative analysis of the positive brown color intensity.

Various experimental groups exhibited changes in iNOS expression. A comparison was made between the group that received cisplatin and the control group. Administering Tri to rats treated with cisplatin resulted in a notable decrease in iNOS expression, especially in the high-dose group. This was confirmed through quantitative analysis of iNOS expression optical density using image analysis software. Each bar indicates the average value plus or minus the standard error of six measurements. The values above the pairwise comparison indicate the precise *P*-value. TRI stands for Trimetazidine, while Cis stands for cisplatin.

## 4. Discussion

The current study provided detailed insights into the positive effects of TRI on various parameters relevant to PN. Rats treated with CIS exhibited alterations in the motor coordination parameters viz., decreased time spent on the rotarod and increased threshold time of Hind paw cold allodynia, threshold force of Randall–Selitto mechanical test and reaction time of the hot plate test, and time of adhesive tape compared to control groups. These results were allied with several previous studies, which have consistently shown that CIS administration leads to impaired motor coordination and nociceptive thresholds in rats using various behavioral tests, viz. rotarod falling time [22, 23], the threshold time of Hind paw cold allodynia [24], threshold force of Randall–Selitto mechanical test [25] and reaction time of the hot plate test [26], and time of adhesive tape removal [23]. These tests evaluate the ability of rats to maintain balance and coordination while performing specific motor tasks.

The underlying mechanisms through which CIS induces these neurological effects are not fully understood. However, several hypotheses have been proposed, including oxidative stress, mitochondrial dysfunction, and inflammation. These mechanisms likely interact to disrupt the normal functioning of motor coordination and nociceptive pathways in the central nervous system (CNS) [27].

However, the main mechanism by which CIS induces PN is mainly due to the DNA adducts within the nucleus of neurons, which can hinder the nucleotide excision repair pathway. This results in accumulating DNA-platinum adducts that cannot be effectively removed. Consequently, proper transcription of ribosomal RNA is disrupted [28].

The influx of CIS into peripheral nerves disrupts neuronal homeostasis, leading to oxidative stress, mitochondrial dysfunction, and impaired DNA repair mechanisms. These events collectively activate the signaling pathways involved in neuropathic pain and neuronal injury [29]. Several studies have shown a reduction in dendritic and spinal complexity in rodents following the administration of CIS [30].

Oxidative stress plays a crucial role in CIPN by generating reactive oxygen species (ROS), which overwhelms endogenous antioxidant defense mechanisms, causing oxidative damage to lipids, proteins, and DNA within peripheral nerves [31]. ROS-induced lipid peroxidation and protein modification contribute to axonal degeneration, demyelination, and impaired neuronal conduction, ultimately manifesting as sensory deficits, motor dysfunction, and autonomic disturbances [32].

Furthermore, neuroinflammation has become a significant factor in CIPN [27]. The release of pro-inflammatory cytokines, such as tumor necrosis factor-alpha (TNF-*α*) and interleukin-1 beta (IL-1*β*), from immune cells and resident glial cells within the peripheral nervous system exacerbates neuronal injury. It promotes the development and maintenance of neuropathic pain [33]. The activation of glial cells, particularly microglia and satellite cells, amplifies the inflammatory response, leading to a self-perpetuating cycle of neuroinflammation and neuronal damage [34].

Trimetazidine, on the other hand, is a pharmaceutical agent with a primary mechanism of action related to cardiac metabolism; evidence suggests that TRI may also affect the CNS. TRI has been used primarily in treating cardiovascular disorders [9]. TRI functions by optimizing the energy metabolism of the heart muscle cells, thereby improving their efficiency and reducing the symptoms of angina. It acts as a metabolic agent, modulating cellular energy production and protecting the heart against ischemic damage [35].

The current study's findings reveal that TRI treatment effectively preserves nerve integrity, as evidenced by improved motor coordination, restored sensory thresholds, and histopathological evidence of reduced nerve damage and enhanced myelination. These observations suggest that TRI is crucial in maintaining the structural and functional integrity of peripheral nerves affected by CIPN. There is limited research specifically investigating the effects of TRI in animal models. However, TRI has been reported to have protective effects against animal studies on the effects of brain ischemia and reperfusion [36]. It enhances brain function by increasing glucose uptake in the brain, preserving the integrity of brain mitochondrial membranes, and safeguarding neuronal cells from intracellular acidosis [37]. Additionally, it elevates the levels of ATP in the CNS [38] and prevents oxidative damage in the hippocampus by upregulating antioxidant enzymes and inhibiting lipid peroxidation in an animal model of Alzheimer's disease [39]. In terms of seizure activity, TRI significantly raises the seizure threshold in the increasing-current electroshock seizure test [40] and prevents the development of seizures in mice [41]. TRI also showed after end-to-end repair in a peripheral nerve injury model, evidenced by the increase in the number of axons [42].

The precise mechanisms underlying the effects of TRI motor coordination and nociceptive thresholds in rats are not yet fully understood. However, it has been suggested that its ability to modulate cellular energy metabolism, reduce oxidative stress, and attenuate inflammation may contribute to its neurological effects [43].

In the current investigation, TRI-treated rats showed amelioration in the neuroinflammation associated with CIPN, evidenced by the suppression of pro-inflammatory cytokine expression and inhibition of glial cell activation in peripheral nerves viz., NF*κ*B and *p*-NF*κ*B. This indicates the anti-neuroinflammatory properties of TRI by modulating neuroinflammatory responses, and TRI contributes to the mitigation of nerve inflammation and subsequent neuropathic symptoms. Moreover, TRI exhibits robust neuroprotective effects by attenuating neuro-oxidative stress [36]. The impact of TRI on oxidative stress parameters in the sciatic nerve during CIPN in rats has been investigated, evidenced by amelioration of sciatic nerve MDA, NO as well as Nrf2.

These findings highlight TRI's ability to counteract the detrimental effects of neuro-oxidative stress, which is a major contributor to nerve damage in CIPN. Several studies have examined the effects of TRI on oxidative stress markers in the sciatic nerve, including levels of ROS, antioxidant enzyme activity, and lipid peroxidation products [44, 45, 46].

Consistent with our findings, Jain et al. [41] attributed the anticonvulsant effects of TRI in mice primarily to its potent antioxidant activity [41]. The well-known antioxidant properties of TRI are believed to be indirectly mediated through the enhancement of antioxidant enzymes [47]. This effect may be linked to its ability to increase the activation of p-AMPK, which plays a significant role in restoring neuronal energy balance [48]. AMPK phosphorylates Nrf2 at the Ser550 residue, which promotes nuclear accumulation of Nrf2 for antioxidant response element (ARE)-driven gene transactivation [49].

Furthermore, TRI has been reported to increase the ATP/ADP ratio in the hippocampus of diabetic epileptic rats [50]. It has also been demonstrated to restore ATP synthesis after inhibition of the cerebral respiratory process of the mitochondria by cyclosporin [51]. The neuroprotective effects of TRI have been well-documented. The capacity to restore glutamate homeostasis in the hippocampus is linked to its effects on the mitochondrial redox state, the ERK1/2 and AMPK signaling pathways, and oxidative stress [43].

mTOR, a well-known autophagy hub protein in cells, is a negative regulator of autophagy. Growing evidence suggests that targeting mTOR could be a promising therapeutic approach for diseases involving autophagy [52]. Furthermore, mTOR is involved in the regulation of cell autophagy in fibrosis-related diseases [53]. The PI3K-mTOR pathway, an established autophagy pathway, controls the processes of cell growth, survival, and movement [54]. The AMPK, involved in regulating energy for cell proliferation, migration, and maintaining stability, is linked to autophagy mechanisms by influencing mTOR modulation [55]. There is evidence to show that NF-*κ*B activity can be controlled through the AMPK-mediated PI3K/mTOR pathway. By decreasing mTOR activity, AMPK activation may dampen NF-*κ*B signaling. The AMPK/mTOR axis inhibits NF-*κ*B activity in many ways. These include the control of NF-*κ*B target gene expression and manipulating I*κ*B, an inhibitor of NF-*κ*B [56].

In rats with cerebral ischemia/reperfusion damage or chronic cerebral hypoperfusion, the treatment of TRI has been demonstrated to enhance motor coordination [36]. More studies are required to understand the underlying processes better and extend the results to diverse situations and pain models. Still, research on its effects on motor coordination and nociceptive thresholds in rats is promising.

Overall, our results provide credence to the neuroprotective effects of TRI in CIPN by attacking several underlying mechanisms simultaneously, such as energy deprivation, neuro-oxidative stress, neuroinflammation, and the integrity of the nervous system. By enhancing motor coordination, nociceptive thresholds, nerve conduction velocities, gene expression levels, oxidative stress parameters, cellular energy metabolism, inflammatory markers, histopathological alterations, and iNOS expression in the sciatic nerve, TRI demonstrates its therapeutic potential for alleviating CIPN.

## 5. Conclusion

**Figure 9:**
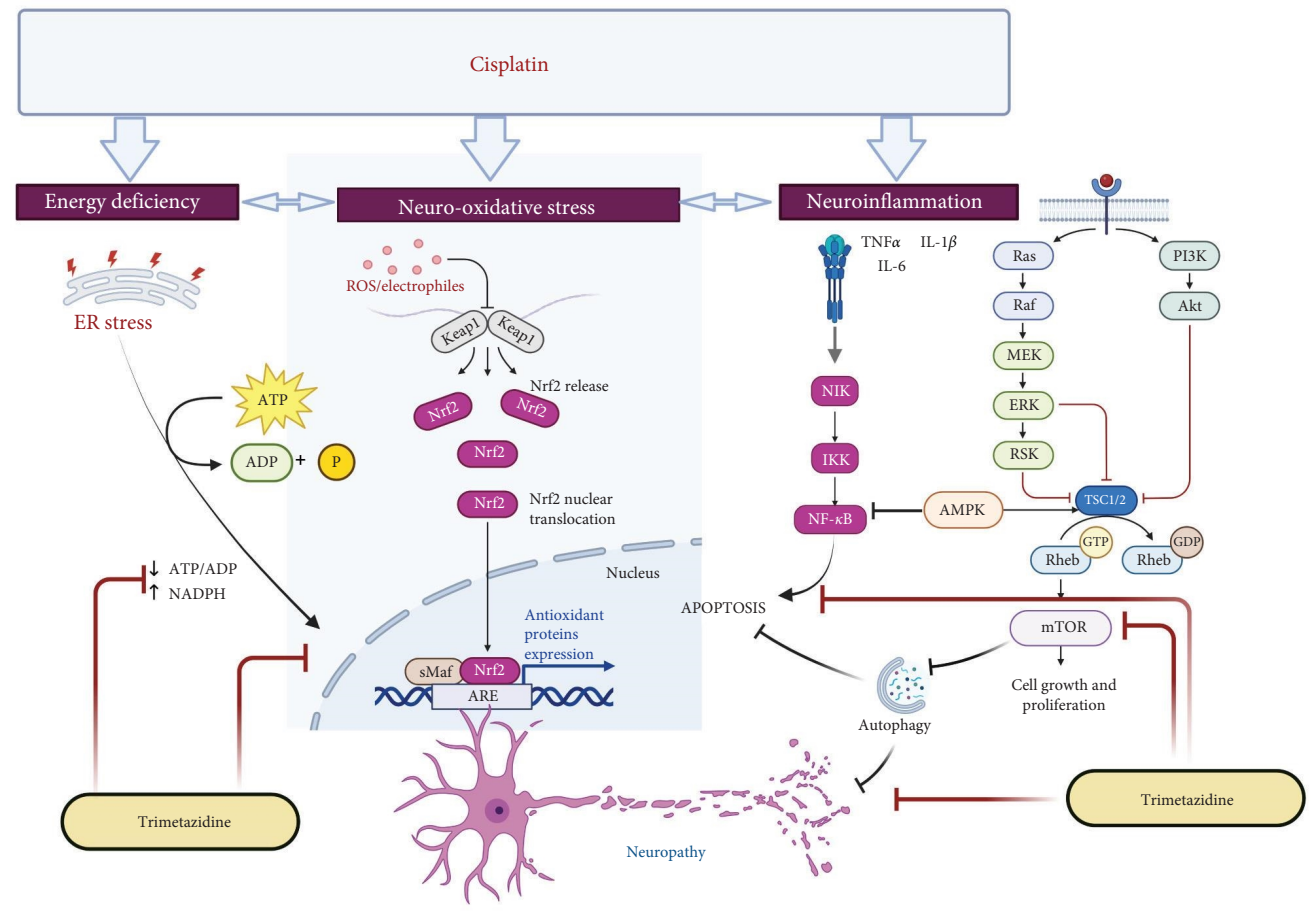
Illustration of trimetazidine associated pathway in suppression Cis-induced peripheral neuropathic rats. SNVC, sensory motor nerve conduction velocity; MNCV, motor nerve conduction velocity, AMPK, AMP-activated protein kinase; mTOR, Mammalian target of rapamycin; PI3K, phosphoinositide 3-kinases; MDA, malondialdehyde; NO, nitric oxide; Nrf2, nuclear factor erythroid 2–related factor 2, ATP, adenosine triphosphate; ADP, adenosine diphosphate; NADPH, nicotinamide adenine dinucleotide phosphate; *p*-NF-*κ*B, phosphate nuclear factor kappa B; NF-*κ*B, nuclear factor kappa B.

In conclusion, our study suggests that TRI has neuroprotective, analgesic, antioxidant, anti-inflammatory, and regenerative properties. TRI effectively restored nerve function, attenuated oxidative stress and inflammation, and promoted histological recovery in the peripheral nerves via the interplay of AMPK-mediated PI3K/mTOR, Nrf2, and NF-*κ*B signaling pathways, as summarized in Figure 9. Furthermore, the therapeutic potential of TRI as a neuroprotective agent in CIPN provides a foundation for future research and clinical applications aimed at improving the quality of life for patients undergoing chemotherapy. Ultimately, the development of TRI as a neuroprotective agent holds promise for improving the quality of life for cancer patients undergoing CIS-based chemotherapy by mitigating the debilitating effects of PN.

## 6. Limitation of the Current Study

Incorporating western blot analysis to measure protein levels and activation states of key components in pathways such as PI3K/Akt/mTOR, AMPK, autophagy (LC3, p62), and fatty acid oxidation (CPT1A, PPAR).

While our study presents promising results regarding the neuroprotective effects of trimetazidine against CIPN, it is essential to acknowledge certain limitations that warrant further investigation; considering the administration of trimetazidine to the normal saline group in another experiment will provide valuable insights into the general effects of trimetazidine on gene expression in a healthy, untreated condition. Further incorporating Western blot analysis to measure protein levels and activation states of crucial components in pathways such as PI3K/Akt/mTOR, AMPK, autophagy (LC3, p62), and fatty acid oxidation (CPT1A, PPAR*α*) in sciatic nerves would provide valuable insights into the modulation of these pathways by trimetazidine.

Also, utilizing specific pharmacological inhibitors or siRNA knockdowns to block pathways like AMPK or mTOR, both in vitro and in vivo, would elucidate whether preventing their activation abolishes the protective effects of trimetazidine, establishing their functional importance. Assessing downstream transcriptional targets of pathways, such as the Nrf2 antioxidant response through quantitative PCR (qPCR), would further delineate the activation of these pathways by trimetazidine.

Besides, conducting metabolomic analysis could identify alterations in energy metabolites and fatty acid oxidation resulting from trimetazidine treatment, providing additional insights into its mechanisms of action. Evaluating autophagic flux to determine trimetazidine's effects on autophagy induction would offer further understanding of its modulation of cellular processes. By addressing these limitations and incorporating additional experiments probing the effects of trimetazidine on PI3K/mTOR, AMPK, autophagy, metabolism, and oxidative stress pathways, to provide a more comprehensive understanding of the mechanisms underlying its neuroprotective effects observed in this CIPN model.

## Data Availability

All relevant data are within the manuscript.
